# Two year mortality and associated factors in a cohort of children from rural Uganda

**DOI:** 10.1186/1471-2458-14-314

**Published:** 2014-04-05

**Authors:** Patrick Nabongo, Suzanne Verver, Elizabeth Nangobi, Ronald Mutunzi, Anne Wajja, Harriet Mayanja-Kizza, Dan Kadobera, Edward Galiwango, Robert Colebunders, Philippa Musoke

**Affiliations:** 1 Infectious Diseases Institute, Kampala, Uganda; 2 KNCV Tuberculosis Foundation, the Hague, Netherlands; 3 Makerere University Iganga/Mayuge Demographic Surveillance Site, Kampala, Uganda; 4 Department of Paediatrics and Child Health, Makerere University College of Health Sciences, Kampala, Uganda; 5 Department of Medicine, Makerere University College of Health Sciences, Kampala, Uganda; 6 Institute of Tropical Medicine, Antwerp, Belgium; 7 University of Antwerp, Antwerp, Belgium; 8 Uganda National TB/Leprosy Program, P.O. Box 16069, Wandegeya, Kampala, Uganda

**Keywords:** Mortality, Infants, Factors associated with mortality, Verbal autopsy

## Abstract

**Background:**

As part of site development for clinical trials in novel TB vaccines, a cohort of infants was enrolled in eastern Uganda to estimate the incidence of tuberculosis. The study introduced several mortality reduction strategies, and evaluated the mortality among study participants at two years. The specific of objective of this sub-study was to estimate 2 year mortality and associated factors in this community-based cohort.

**Methods:**

A community based cohort of 2500 infants was enrolled from birth up to 8 weeks of age and followed for 1–2 years. During follow up, several mortality reduction activities were implemented to enhance cohort survival and retention. The verbal autopsy process was used to assign causes of death.

**Results:**

A total of 152 children died over a median follow up period of 2.0 years. The overall crude mortality rate was 60.8/1000 or 32.9/1000 person years with 40 deaths per 1000 for children who died in their first year of life. Anaemia, malaria, diarrhoeal diseases and pneumonia were the top causes of death. There was no death directly attributed to tuberculosis. Significant factors associated with mortality were young age of a mother and child’s birth place not being a health facility.

**Conclusion:**

The overall two year mortality in the study cohort was unacceptably high and tuberculosis disease was not identified as a cause of death. Interventions to reduce mortality of children enrolled in the cohort study did not have a significant impact. Clinical trials involving infants and young children in this setting will have to strengthen local maternal and child health services to reduce infant and child mortality.

## Background

In Uganda as in many parts of the developing world, infant and child mortality are a public health concern. According to the last Uganda demographic and health survey (UDHS) report, infant and under-five mortality rates were 76/1000 and 137/1000 live births respectively [1]. In the east-central region of the country, where our study was located, the infant and under-five mortality rates were 74/1000 and 128/1000 [1]. The leading direct causes of infant and under-five mortality in Uganda include malaria accounting for 25%, neonatal diseases 23%, pneumonia 19%, diarrhoeal disease 17%, and AIDS 6% [2]. However, very little is known about the contribution of tuberculosis to mortality in early childhood.

A community based cohort of 2500 children was enrolled from birth up to 8 weeks of age and followed for 1–2 years in a demographic surveillance site in rural Eastern Uganda to estimate incidence of tuberculosis. This cohort was part of a community based epidemiological study to build capacity for future evaluation of novel TB vaccines. During follow up, the study implemented several mortality reduction activities including direct financial and transportation support for medical care for severely sick children to enhance cohort survival and retention. During the main study, we conducted a sub-study to estimate mortality rate and describe causes and associated factors for mortality among this cohort.

## Methods

### Study setting and population

The study was done in the Makerere University demographic surveillance site (DSS) located in the southeastern part of the country about 120 Km from the capital city, Kampala. The study area covered seven sub-counties and about 120 villages that straddle two districts of Iganga and Mayuge. The DSS has a population of 130,000 with approximately 19,000 households. Routine household socio-demographic data was collected from the DSS area including births and deaths.

### Data sources

#### Objectives, procedures and activities of the parent study

The primary objective of the main study was to estimate the incidence of TB disease in the infant cohort. Infants met eligibility criteria for enrollment if they were vaccinated with BCG within 30 days of birth, were ≤ 8 weeks of age and were residents of the DSS area. Eligible infants were consecutively enrolled by convenience sampling until the sample size of 2500 was achieved. Recruitment and enrollment was done at health facilities or at home through village scouts between November 2008 and November 2009. Study follow up was for a maximum of two years.

Participants were assessed at enrolment and during study follow-up at day 42, 84, 122, 243, 365, 487, 609 and 730 from the enrolment date. History of symptoms suggestive of TB, household contact with a TB patient and PPD skin testing were used to screen for TB. Those suspected of TB disease were referred to a case verification ward for diagnostic work-up. The diagnostic work up for tuberculosis included a CXR, induced sputum and gastric aspirates for Ziehl Neelsen stain and culture.

### Mortality reduction strategies

Mortality reduction interventions were introduced about six months into the study and were implemented at weekly study follow-up clinics. We used the integrated management of childhood illnesses (IMCI) guidelines to provide clinical care to all sick participants seen during follow up. All participants received insecticide treated mosquito bed nets at enrolment or within six weeks of enrolment. Iron supplementation was provided to all participants for at least one month during follow up. All participants were de-wormed every six months from the age of six months. Participants with severe illness received financial support for medical care and transportation to referral facilities and those with acute severe malnutrition detected at the weekly follow-up clinics were linked to a local hospital where they received ready-to-use feeds (plumpy nut paste). A number of the activities mentioned above should be part of the normal standard of care but are often not implemented.

### Death identification and cause assignment

Deaths of child participants were identified by trained DSS village volunteers/scouts or by the study field staff during follow-up. Upon notification of the study team, trained DSS field workers conducted a formal verbal autopsy within a period of six weeks to one year after death notification. A verbal autopsy tool adopted from INDEPTH/WHO which was adapted to capture parameters of tuberculosis was completed [3]. The form was then reviewed by three independent medical officers who assigned cause of death by use of the international classification of diseases (ICD-10) criteria. Cause of death was assigned when at least two medical officers concurred in their opinions or else, it was assigned as undetermined.

### Data analysis

Causes of death and characterization of deaths were analyzed using Excel. We used STATA 11 to analyze baseline participant characteristics and comparison between the dead and the surviving participants was made by a chi-square statistic. Person years of observation (Pys) for each participant were calculated by subtracting the date of entry into the study from the last date of contact with the participant divided by 365 days. For those participants that died, the date of death was taken as the last date of contact while for those that survived, the last date of follow-up provided the last contact date. We used Cox proportional hazard model to analyze for factors associated with mortality.

### Ethics and regulatory considerations

The study was conducted in compliance with the Declaration of Helsinki [4], Protection of human Volunteers (21 CFR 50), Institutional Review Boards (21 CFR 56), and Obligations of Clinical Investigators (21 CFR 312). The protocol for the main study was reviewed and approved by the Makerere University School of Public Health (MUSPH) Ethics Committee; by the Aeras (a US based TB Vaccine Foundation) Institutional Review Board and by the Uganda National Council of Science and Technology (UNCST). Written informed consent was obtained from all study participants prior to enrolment into the study and in accordance with the Declaration of Helsinki (21 CFR 50.25) and local research regulations.

## Results

### Baseline participant and parent’s characteristics

A cohort of 2500 infants was enrolled with a median age of 19 days (range: 0–56 days) and was followed up for a median duration of 2.0 years. A total of 152 (6%) infants died and the population of the dead participants was generally similar to the surviving ones except with respect to age of the mother, father’s education and birth place of participant (see Table 1). Slightly over 50% of the children who died were born outside a health facility. Among the deaths, children born to mothers aged 13–17 years had the highest proportion of deaths (17.2%) while the lowest proportion was in children born to mothers aged ≥40 years (2.5%). With regard to the education level of the parents, the highest proportion of deaths was in children born to parents with no formal education (8.3% for mothers and 10.3% for fathers). Children born to parents with University/tertiary education had the lowest proportion of deaths (4.9% for mothers and 2.8% for fathers). See Table 1.

**Table 1:** Participant and parental baseline characteristics (N = 2500)

	**Dead (n = 152)**		**Live (n = 2348)**	
**Characteristic**	**No**	**%**	**No**	**%**
**Sex** (p-value 0.96)				
Male	79	52.0	1208	51.8
Female	73	48.0	1125	48.2
**Place of birth** (p-value 0.038*)				
Health facility yes	71	46.7	1300	55.4
Not at health facility No	81	53.3	1048	44.6
**Mode of delivery** (p 0.095)				
Caesarian/Assisted	1	0.7	70	3.0
Normal	151	99.3	2278	97.0
**Birth complications** (p 0.64)				
Yes	3	2.0	61	2.6
No	149	98.0	2287	97.4
**History of household TB contact at Day 0** (p 0.54)				
Yes	4	2.6	84	3.6
No	148	97.4	2264	96.4
**Mother’s education level** (p 0.53)				
None	26	17.1	312	13.3
Primary	91	59.9	1502	64.0
Secondary	32	21.1	473	20.1
University/Tertiary	3	2.0	61	2.6
**Mother’s age** (p < 0.001*)				
13-17 yrs	20	13.2	116	4.9
18-24 yrs	60	39.5	1044	44.5
25-29 yrs	27	17.8	507	21.6
30-34 yrs	30	19.7	390	16.6
35-39 yrs	9	5.9	165	7.0
40+ yrs	1	0.7	40	1.7
Unknown	5	3.3	86	3.7
**Mother’s occupation** (p o.11)				
Occupation of direct income	3	2.0	114	4.9
No direct income	149	98.0	2234	95.1
**Father’s age** (p 0.217)				
13-17 yrs	2	1.3	7	0.3
18-24 yrs	27	17.8	387	16.5
25-29 yrs	28	18.4	466	19.9
30-34 yrs	20	13.2	408	17.4
35-39 yrs	20	13.2	355	15.1
40+ yrs	51	33.6	695	29.6
Unknown	4	2.6	30	1.3
**Father’s education level** (p 0.020*)				
None	25	16.5	244	10.4
Primary	71	46.7	1188	50.6
Secondary	52	34.2	775	30.0
University/Tertiary	4	2.6	141	6.0
**Father’s occupation** (p 0.281)				
Direct income	48	31.6	843	35.9
No direct income	104	68.4	1505	64.1

Note: The dead participants were significantly different from the surviving ones in respect of mother’s age, place of birth and father’s education.

### Health seeking behaviour

Formal medical care was sought in 135 of the 152 deaths (88.8%). Among the 135 cases where care was sought, the first point of care was a government/formal health facility in 61 cases (45.2%). Referral to hospital from first point of care was recommended for 70 children. However, nearly a third (20) of them failed to go for referral care and died at home. Overall, only about 40% (60) of the deaths happened in a health facility.

### Mortality and causes of mortality

**Figure 1:**
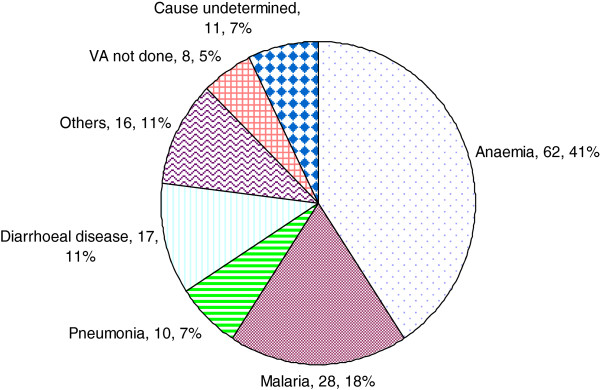
**Likely causes of death by verbal autopsy.** The figure shows that anaemia, malaria, diarrhoeal disease and pneumonia were the 4 top single causes of death accounting for 77% of the deaths.

Among the 152 deaths, 65.8% occurred in their first year of life, one of which was a death in the neonatal period due to neonatal septicaemia and 31.6% after the first year of life. The age at death for 4 participants was undetermined (Table 2). The total follow up person time for this cohort was 4621.7 person years. The observed overall crude mortality rate was 32.9/1000 Person years or 60.8/1000 children while the rate for children who died in their first year of life among this cohort was 40/1000 (Table 3). With regard to cause of death; anaemia malaria; pneumonia and diarrhoea were the top causes of death, accounting for 77% of deaths. See Figure 1.

**Table 2:** Likely causes of death by age-at-death (in days)

**Causes of death**	**0-28**	**>28-180**	**>180-365**	**>365-730**	**>730**	**Age Unkn**	**Total**
	**No**	**No**	**No**	**No**		**No**	**No**
Severe anaemia	0	12	25	22	1	2	62
Malaria with others	0	5	13	9	0	1	28
Pneumonia	0	3	3	3	0	1	10
Diarrhoeal diseases	0	5	9	3	0	0	17
Others**	1	7	3	5	0	0	16
Undetermined	0	5	3	3	0	0	11
*VA not done	0	3	3	1	1	0	8
**Total**	**1**	**40**	**59**	**46**	**2**	**4**	**152**

*Verbal autopsy.

**Others included Diabetes, burns, central nervous system disorders, tetanus, meningitis, poisoning, malnutrition, prematurity, road traffic accident and cardiovascular disorders. The cause of death for one death that occurred during the neonatal period was neonatal septicaemia.

**Table 3:** Mortality rate by background characteristics

**Characteristic**	**n/N**	**Pyrs**	**Mortality rate (Per 1000Pys)**
**Overall**	**152/2500**	**4621.7**	**32.9**
**Gender**			
Male	79/1294	2390.4	33
Female	73/1206	2231.3	32.7
**Mother’s age (Yrs)**			
13-17	20/136	235.1	85.1
18-24	60/1104	2027.2	29.6
25-29	27/534	1001.2	27
30-34	30/420	777.7	38.6
35-39	9/174	323.8	27.8
≥40	1/41	81.3	12.3
**Father’s age (Yrs)**			
13-17	2/9	16.6	120.5
18-24	27/414	735.8	36.7
25-29	28/494	918.1	30.5
30-34	20/428	813.3	24.6
35-39	20/375	689.4	29
≥40	51/746	1383.8	36.9
**Mother’s education**			
None	25/331	612.3	40.8
Primary	91/1593	2948.8	30.9
Secondary	32/505	929.5	34.4
University/Tertiary	3/64	119.1	25.2
**Father’s education**			
None	7/190	350.1	48.6
Primary	71/1259	2345.3	30.3
Secondary	52/827	1517	34.3
University/Tertiary	4/145	266.8	15
**Birth place**			
Hospital/Clinic	71/1371	2542.4	27.9
Other place (TBA, Home, etc.)	81/1129	2079.2	39
**Birth complications**			
**Asphyxia**			
Yes	3/41	71.4	42
No	149/2459	4550.2	32.7
**Congenital anomalies**			
Yes	0/5	7.4	0.0
No	152/2499	4614.2	32.9
**Septicaemia**			
Yes	0/2	4.1	0.0
No	152/2498	4617.6	32.9
**Anaemia**			
Yes	0/1	2.0	0.0
No	152/2499	4619.7	32.9

Pyrs - Person years.

### Associated factors of mortality

#### Mortality by background characteristics and modeling associated factors for mortality

Participants born to mothers in the age groups of 13 – 17 years and ≥40 years showed the highest and lowest mortality rates of 85/1000pys and 12.3/1000pys, respectively (Table 3). Multivariable Cox proportional hazard model revealed young age of a mother and child’s place of birth being outside a health facility as the only significant factors associated with child death (Table 4).

**Table 4:** **Multivariable Cox proportional hazard model with 95% confidence interval and corresponding *P *** **values, of infant deaths in the first 2 year period of follow up**

**Characteristic**	**Adjusted hazard rate ratio (95% CI)**	**P value**
**Gender**		
Male	Referent (1.00)	
Female	0.98 (0.87,1.10)	0.739
**Mother’s age (Yrs)**		
13-17	Referent (1.00)	
18-24	0.56 (0.44,0.72)*	<0.001
25-29	0.56 (0.42,0.74)*	<0.001
30-34	0.58 (0.43 ,0.77)*	<0.001
35-39	0.58 (0.41,0.82)*	0.002
≥40	0.41 (0.24,0.71)*	0.001
**Fathers age**		
13-17	Referent (1.00)	
18-24	2.09 (0.67,6.6)	0.208
25-29	2.11 (0.67,6.67)	0.203
30-34	1.80 (0.57,5.71)	0.319
35-39	2.08 (0.65,6.62)	0.216
≥40	2.03 (0.64,6.42)	0.230
**Mothers education**		
None	Referent (1.00)	
Primary	0.92 (0.77,1.10)	0.361
Secondary	0.94 (0.76-1.17)	0.596
University/Tertiary	0.99 (0.66,1.50)	0.964
**Father’s education**		
None	Referent (1.00)	
Primary	0.99 (0.79,1.26)	0.963
Secondary	1.13 (0.89,1.44)	0.320
University/Tertiary	1.23 (0.74,2.06)	0.558
**Birth place**		
Hospital/Clinic	Referent (1.00)	
Other place (TBA, Home, etc.)	1.13 (1.00,1.28)*	0.034
**Birth complications**		
**Asphyxia**		
Yes	Referent (1.00)	
No	1.24 (0.74,2.06)	0.414

*Characteristics that were observed to be statistically significant.

## Discussion

In this cohort of 2500 infants, 152 participants died with nearly two thirds of them dying in their first year of life. Severe anaemia, malaria, diarrhoeal disease and pneumonia were the top causes of death.

### Mortality

The overall two year crude mortality and the study infant mortality rate for this cohort were 60.8/1000 and 40/1000 respectively. However, the study infant mortality rate was much lower than the infant mortality (IMR) of 74/1000 expected in the region because the study missed most of the neonatal deaths (the median cohort enrolment age was 19 days). Findings from previous studies done in Uganda have shown that neonatal deaths constitute 40% of all infant deaths [1]. This is supported by a situation analysis of newborn health in Uganda in which neonatal mortality rate was estimated at 29/1000 vis-à-vis an IMR of 76/1000 [5]. By extrapolation therefore, this would put the actual IMR (including neonatal deaths) for this cohort at about 69/1000 live births which though slightly lower, is not significantly different from the overall country and region rates of 76/1000 and 74/1000 respectively [1]. This probably indicates that the mortality reduction activities that were implemented in the study participants did not have much effect on the overall child mortality in this cohort. The 2 year crude mortality cannot be compared to under-five mortality rates, since we did not follow the infants until age of five.

### Causes of mortality

Our results showed anaemia, malaria, diarrhoeal diseases and pneumonia to be the leading causes of death in this cohort. This follows a pattern similar to that observed nationally [2]. There were no deaths directly attributable to tuberculosis except for one death where TB was assigned as a probable underlying cause of death. This contrasts with findings from a hospital based descriptive necropsy study done in Zambia in which TB accounted for 11% of respiratory infection deaths in the HIV-infected and 17% in the HIV-negative children aged 0 – 17 months [6]. The higher number of TB cases identified in the Zambia study was probably because it was hospital based. Secondly, the difference could be in the method used to investigate the cause of death which included an autopsy in the Zambian study. It is possible that we under-diagnosed tuberculosis in our study cohort, although we did very thorough investigations that are usually not done in this setting.

Anaemia is common among Ugandan children as indicated by findings from a study done in Uganda which showed that 73% of children aged 6–59 months have anaemia. The study further indicated that in the eastern region of Uganda, 80% of children in this age group had anaemia [2]. These findings give an indication of the burden of anaemia among infants and children in our study area. Similar to what has been reported previously, anaemia was the top cause of death in our study cohort. The underlying cause of anaemia has largely been assumed to be malaria, since most of Uganda is malaria endemic. However, in another study done on iron deficiency anaemia in Uganda, iron deficiency was found to be a significant underlying cause for anaemia in this age group. It was estimated that nearly half of the anaemia in children is due to iron-deficiency and the other half due to other causes such as malaria and worm infestation [2,7].

### Health seeking behaviour

Most of the children who died sought care from a health facility for the sickness that immediately preceded their death. The first point of care for nearly half of them was a local private facility. This is consistent with findings from studies done in rural Kenya on health care seeking behaviour [8,9]. In our study, the occurrence of private providers as first point of care may have been partly because the study ran follow-up clinics only once a week. In these settings, most of the local private providers are not sufficiently equipped to handle life threatening child conditions. Consequently, this practice creates delay in accessing appropriate care and increases risk of death.

With regard to child’s place of death, we found that most deaths did not happen in a health facility. This is in spite of the fact that about 90% of children who died had sought care from a health facility for the sickness that preceded their death. This is similar to findings of a prospective cohort study done in rural Malawi [10]. This has implications for child health services and for clinical research involving children. Among those who sought care, about half of them were referred to hospital by their first point of care. However, one third of those referred did not reach the referral care points. Clinical research studies intended for this setting need to take this into consideration as they plan for follow up.

### Factors associated with mortality

The dead and surviving participants had similar baseline socio-demographic and clinical factors except for mother’s age, father’s education and place of birth. Children born to very young mothers (13–17 yrs) had the highest risk of dying. This is consistent with other reports and is related to the fact that younger mothers tend to have lower education and socio-economic characteristics which contribute to the higher infant mortality [11]. The risk of dying generally diminished with increasing maternal age. This decrease in risk of mortality with increase in maternal age is probably due to the expected accumulation of child-care experience with increasing maternal age. Available literature supports this finding, but other studies show that children born to mothers aged 40 years and above are also at higher risk of dying [12,13]. However in our study, the likelihood of dying was lowest for children born to mothers aged 40 years and above. The relatively small number of children in this cohort born to mothers aged 40 years and above could explain this unusual finding.

Other risk factors for infant and child mortality reported elsewhere include mother’s death during infant’s first year of life, death of previous sibling, being a twin, parental education, birth spacing, distance to nearest health unit, season of birth, etc [14-16]. In our cohort parent’s education level was surprisingly insignificant although findings from previous studies have shown parental education to have significant and negative relationship with child mortality [17-19]. Fathers with higher education levels are likely to have skilled occupations that lead to lower child mortality rates because of their higher socio-economic conditions [19]. Concerning child’s place of birth, in our cohort children born in a health facility had a significantly lower risk of dying than those whose place of birth was outside a health facility. In other studies however, child’s place of birth has not been found to be of significant influence [12,20,21].

### Limitations

Due to limitation in the scope of our study, we were unable to measure the effect that other known child mortality determinants had on our cohort. Analysis of risk factors for death was applied to the entire sub-cohort of the dead children without segregating between those who died in their first year of life and those who died after their first year of life, due to low numbers. This is a critical limitation because risk factors for death in the first year of life are different from those after the first year of life. We implemented child mortality reduction activities for all our participants but due to lack of a comparison group, we were unable to reasonably assess the effect of these activities on mortality measurements. A verbal autopsy was done at least six weeks after death of a participant. This could have affected the ability of a parent/guardian to recall events that led to the death of their child.

## Conclusion

The overall crude mortality in our cohort of children was 60.8/1000 live births which was unacceptably high for a future vaccine trial site. Interventions to reduce mortality of children enrolled in the cohort study did not have a significant impact. In this cohort, deaths were mainly due to common childhood illnesses but not tuberculosis. Vaccine clinical research studies involving children less than five years of age, intended for this setting, will need to consider implementing child survival interventions to reduce child mortality.

## Abbreviations

AIDS: Acquired immunodeficiency syndrome; DSS: Demographic surveillance site; EDCTP: European and developing countries clinical trials partnership; FANTA: Food and Nutrition Technical Assistance; HIV: Human immunodeficiency virus; ICD: International classification of diseases; IMCI: Integrated management of childhood illnesses; IMR: Infant mortality rate; INDEPTH: International Network of Demographic Surveillance Sites; PPD: Purified protein derivative; PY: Person years; TB: Tuberculosis; UDHS: Uganda Demographic and Health Survey; UBOS: Uganda Bureau of Statistics; WHO: World Health Organization.

## Competing interests

The authors declare that they have no competing interests.

## Authors’ contributions

PN and AW participated in the designing and coordination of the study, and were part of the project team for review of study manuscripts including this one. Being the lead Author, PN made a major contribution in drafting this paper. Authors EN, RM, DK and EG participated in study coordination, data collection, data analysis and were part of the project team for review of all study manuscripts; this one inclusive. PM, HMK, SV and RC were critical members of the team that conceived the study. They guided the study, contributed to data interpretation and were key members of the project review team during drafting of this manuscript. Authors PM and HMK held overall responsibility of the study. All authors read and approved this manuscript. PM gave the final approval.

## Pre-publication history

The pre-publication history for this paper can be accessed here:

http://www.biomedcentral.com/1471-2458/14/314/prepub
